# Bathing Frequency and Onset of Functional Disability Among Japanese Older Adults: A Prospective 3-Year Cohort Study From the JAGES

**DOI:** 10.2188/jea.JE20180123

**Published:** 2019-12-05

**Authors:** Akio Yagi, Shinya Hayasaka, Toshiyuki Ojima, Yuri Sasaki, Taishi Tsuji, Yasuhiro Miyaguni, Yuiko Nagamine, Takao Namiki, Katsunori Kondo

**Affiliations:** 1 Graduate School of Medicine, Chiba University, Chiba, Japan; 2 Department of Japanese Oriental “Kampo” Medicine, Chiba University, Chiba, Japan; 3 Faculty of Human Life Sciences, Tokyo City University, Tokyo, Japan; 4 Department of Community Health and Preventive Medicine, Hamamatsu University School of Medicine, Shizuoka, Japan; 5 Department of International Health and Collaboration, National Institute of Public Health, Saitama, Japan; 6 Center for Preventive Medical Sciences, Chiba University, Chiba, Japan; 7 Center for Gerontology and Social Science, National Center for Geriatrics and Gerontology, Aichi, Japan; 8 Center for Well-being and Society, Nihon Fukushi University, Aichi, Japan

**Keywords:** tub bathing, functional decline, care, older people, prevention

## Abstract

**Background:**

While bathing styles vary among countries, most Japanese people prefer tub bathing to showers and saunas. However, few studies have examined the relationship between tub bathing and health outcomes. Accordingly, in this prospective cohort study, we investigated the association between tub bathing frequency and the onset of functional disability among older people in Japan.

**Methods:**

We used data from the Japan Gerontological Evaluation Study (JAGES). The baseline survey was conducted from August 2010 through January 2012 and enrolled 13,786 community-dwelling older people (6,482 men and 7,304 women) independent in activities of daily living. During a 3-year observation period, the onset of functional disability, identified by new certification for need of Long-Term Care Insurance, was recorded. Tub bathing frequencies in summer and winter at baseline were divided into three groups: low frequency (0–2 times/week), moderate frequency (3–6 times/week), and high frequency (≥7 times/week). We estimated the risks of functional disability in each group using a multivariate Cox proportional hazards model.

**Results:**

Functional disability was observed in a total of 1,203 cases (8.7%). Compared with the low-frequency group and after adjustment for 14 potential confounders, the hazard ratios of the moderate- and high-frequency groups were 0.91 (95% confidence interval [CI], 0.75–1.10) and 0.72 (95% CI, 0.60–0.85) for summer and 0.90 (95% CI, 0.76–1.07) and 0.71 (95% CI, 0.60–0.84) for winter.

**Conclusion:**

High tub bathing frequency is associated with lower onset of functional disability. Therefore, tub bathing might be beneficial for older people’s health.

## INTRODUCTION

Population aging is a critical issue in most developed and developing countries.^1^^,^^2^ With a proportion of individuals 65 years or older of 27.8% (in August 2017), Japan has the most aged population in the world.^3^ This proportion is expected to increase rapidly in the near future.^4^ A growing number of older people have functional disability requiring care in daily life, and there are increasingly fewer young people to support them. Therefore, prevention of functional disability is an important issue for public health in Japan and other counties.

Although the Japanese have one of the longest life expectancies in the world,^4^ the reasons for their longevity are not well understood. The Japanese diet^5^ and social cohesion^6^^,^^7^ appear to play some part. Some other aspects of the Japanese lifestyle might also have a protective effect on health.

The Japanese prefer to take baths, especially in a bathtub, rather than shower or sauna bath, not only for cleanliness, but also to feel warm and refreshed and to aid sleep.^8^^,^^9^ The relationship between bathing and health outcomes has been reported in several studies. In cross-sectional studies, tub bathing frequency was reported to be associated with good sleep quality, low perceived stress, and good self-rated health.^10^^–^^12^ Only one longitudinal study in Japan, with a 5-year observational period, has examined this issue,^13^ finding that tub bathing frequency was inversely associated with the onset of functional disability. However, that study was limited in generalizability because it specifically enrolled outpatients and had a relatively small sample size (*n* = 610). In a large cohort study in Finland, Laukkanen et al^14^ identified a strong negative association between sauna bathing frequency and cardiovascular and all-cause mortality, although sauna bathing is clearly distinct from tub bathing.

Here, we conducted a large prospective cohort study targeting the general population to evaluate the association between tub bathing frequency and the onset of functional disability among Japanese older adults.

## METHODS

### Study population and setting

We used data from the Japan Gerontological Evaluation Study (JAGES).^15^ The baseline survey was performed from August 2010 through January 2012. The target population was community-dwelling older adults, aged 65 or older, not certified to need care under Long-Term Care Insurance (LTCI), and living in 18 municipalities in eight prefectures in Japan. A total of 110,447 randomly selected people were mailed self-report questionnaires asking about their health status, habits, and lifestyle; 72,760 completed questionnaires were returned (response rate, 65.9%). For 69,408 of them (95.4%), we were able to refer to the LTCI database, which contains the information necessary for follow-up. In a randomly selected one-fourth or one-fifth of the questionnaires (the rate varied depending on municipality), participants were asked about bathing habits (*n* = 16,416). We excluded individuals who were not independent in activities of daily living (ADL) (*n* = 896) or missing information on bathing frequency in summer or winter (*n* = 1,734). This left 13,786 individuals (6,482 men and 7,304 women) for analysis; their mean age was 73.4 (standard deviation, 6.0) years.

We observed the participants for 3 years from the baseline survey and recorded the onset of functional disability (defined in the next section), movement out of the municipality, and death. This information was obtained from the LTCI database after the end of the observational period; this database is maintained by the local authority of each municipality.

This study was approved by the human research ethics committee of Nihon Fukushi University (No. 10-05). All individuals enrolled in the baseline survey were informed that their participation in the study was voluntary and that completion and return of the questionnaire indicated their consent to participate.

### Outcome

We defined functional disability as being dependent in ADL by physical or cognitive difficulty, which was identified by certification of need for LTCI (including the “need support” level).^16^^–^^18^ The Japanese LTCI was established to improve older people’s welfare by promoting public care. LTCI is a compulsory coverage insurance, and its benefits are obtained by older people when they apply and are certified to need care. The certification is standardized and based on information on the applicant’s ADL gathered by a qualified investigator and on comments from the family physician.^18^

### Exposure

In the questionnaire, participants were asked, “How many times a week do you take a bath in a bathtub in summer and in winter?” The original question is shown in eFigure 1. We divided tub bathing frequencies into three groups: low frequency (0–2 times/week), moderate frequency (3–6 times/week), and high frequency (≥7 times/week) for both summer and winter.^11^^,^^12^

In the Japanese style of bathing, the water temperature is usually 39–42°C. Bathers tend to spend 5–15 minutes in the bathtub, soaking deeply up to the shoulder level.^19^ Bathing is usually performed in the late evening.^9^ Because changes in the seasons are clear in Japan (hot and wet in summer and cold and dry in winter),^20^ the associations between bathing and health outcomes were expected to differ among seasons. Therefore, we measured tub bathing frequency separately in summer and winter.

### Covariates

In the baseline survey, we recorded participants’ demographic factors (age, sex, and marital status) and asked about socioeconomic status (employment, equivalized income, and years of education), heath-related behaviors (smoking status, alcohol consumption, and body mass index), and self-reported health status (treatment for any disease, physical strength, cognitive function, depression, and instrumental ADL).

These variables were divided into the following categories: age (65–69, 70–74, 75–79, 80–84, or ≥85 years old), sex (male or female), marital status (married or single), employment status (not employed or employed), equivalized income (≤1.99, 2.00–3.99, or ≥4.00 million yen/year), years of education (0–9, 10–12, or ≥13 years), smoking status (never smoker, former smoker, or current smoker), alcohol consumption (nondrinker or drinker), body mass index (≤18.4, 18.5–24.9, or ≥25.0 kg/m^2^), treatment for any disease (without any disease or with any disease), physical strength (normal or low), cognitive function (normal or a decline), depression (not depressed or depressed), and instrumental ADL (independent or dependent). Physical strength and cognitive function were assessed based on the Kihon Checklist,^21^^,^^22^ which was developed to identify older people who are at risk of functional disability. Depression was assessed by the shorter version of the Geriatric Depression Scale,^23^ which includes 15 questions, with a cutoff point of ≥5 indicating “depressed”. Instrumental ADL was evaluated using the Tokyo Metropolitan Institute of Gerontology Index of Competence,^24^ which consists of five questions; participants who missed ≥1 point were regarded as being “dependent”.

### Statistical analysis

After describing the baseline characteristics of the study participants, we used a multivariate Cox proportional hazards model to estimate the risks of functional disability according to tub bathing frequency. In the multivariate-adjusted model, we adjusted for 14 covariates: age, sex, marital status, employment, equivalized income, years of education, smoking status, alcohol consumption, body mass index, treatment for any disease, physical strength, cognitive function, depression, and instrumental ADL. All of these covariates were assumed to be potential confounders. If the information on a covariate was missing, we classed the participant into a “missing” category when performing the analysis. Participants who moved out or died without functional disability were censored.

Next, we performed sensitivity analysis in the multivariate-adjusted model, excluding participants whose follow-up period was less than 1 year in order to take account of non-observable risk factors. Subgroup analysis was then performed with stratification by sex, age (65–74 or ≥75 years old), and baseline health status, such as treatment for any disease, physical strength, cognitive function, depression, and instrumental ADL. When performing this analysis, we used the variables in the multivariate-adjusted model except for the variable used for the stratification.

The proportional hazards assumption was graphically verified by plotting the log [−log] transformation of the cumulative survival curve of each exposure group.^25^

We used SPSS Statistics version 24.0 (IBM Inc., Armonk, NY, USA) for all analyses. A *P*-value less than 0.05 was considered significant.

## RESULTS

Baseline characteristics of the study participants are described in Table 1. The numbers of individuals in each bathing frequency group (low, moderate, and high) were 1,448, 2,777, and 9,561 for summer and 1,347, 4,021, and 8,418 for winter, respectively. Compared with the other groups, the people classed in the high-frequency group were younger and more likely to be married, not depressed, and independent in instrumental ADL and have a moderate-to-high equivalized income, normal physical strength, and normal cognitive function. This tendency was clearer in winter than in summer.

**Table 1.** Baseline characteristics of study participants according to the frequency of tub bathing in summer and in winter

Variables	Summer						Winter					
	Frequency of tub bathing, times/week						Frequency of tub bathing, times/week					
	
	0–2		3–6		≥7		0–2		3–6		≥7	
	*n*	(%)	*n*	(%)	*N*	(%)	*n*	(%)	*n*	(%)	*N*	(%)
Participants	1,448		2,777		9,561		1,347		4,021		8,418	
Age, years												
65–69	469	(32.4)	785	(28.3)	3,209	(33.6)	285	(21.2)	1,172	(29.1)	3,006	(35.7)
70–74	394	(27.2)	820	(29.5)	2,766	(28.9)	345	(25.6)	1,197	(29.8)	2,438	(29.0)
75–79	305	(21.1)	616	(22.2)	2,038	(21.3)	331	(24.6)	887	(22.1)	1,741	(20.7)
80–84	194	(13.4)	373	(13.4)	1,083	(11.3)	256	(19.0)	539	(13.4)	855	(10.2)
≥85	86	(5.9)	183	(6.6)	465	(4.9)	130	(9.7)	226	(5.6)	378	(4.5)
Sex												
Male	732	(50.6)	1,395	(50.2)	4,355	(45.5)	699	(51.9)	1,806	(44.9)	3,977	(47.2)
Female	716	(49.4)	1,382	(49.8)	5,206	(54.5)	648	(48.1)	2,215	(55.1)	4,441	(52.8)
Marital status												
Married	911	(62.9)	1,798	(64.7)	6,880	(72.0)	812	(60.3)	2,518	(62.6)	6,259	(74.4)
Single	452	(31.2)	850	(30.6)	2,341	(24.5)	452	(33.6)	1,319	(32.8)	1,872	(22.2)
Missing	85	(5.9)	129	(4.6)	340	(3.6)	83	(6.2)	184	(4.6)	287	(3.4)
Employment												
Not employed	969	(66.9)	1,895	(68.2)	6,368	(66.6)	921	(68.4)	2,734	(68.0)	5,577	(66.3)
Employed	297	(20.5)	511	(18.4)	2,069	(21.6)	212	(15.7)	764	(19.0)	1,901	(22.6)
Missing	182	(12.6)	371	(13.4)	1,124	(11.8)	214	(15.9)	523	(13.0)	940	(11.2)
Equivalized income, million yen/year												
Low (≤1.99)	598	(41.3)	1,299	(46.8)	3,546	(37.1)	619	(46.0)	1,820	(45.3)	3,004	(35.7)
Middle (2.00–3.99)	428	(29.6)	788	(28.4)	3,244	(33.9)	336	(24.9)	1,170	(29.1)	2,954	(35.1)
High (≥4.00)	112	(7.7)	148	(5.3)	1,079	(11.3)	70	(5.2)	247	(6.1)	1,022	(12.1)
Missing	310	(21.4)	542	(19.5)	1,692	(17.7)	322	(23.9)	784	(19.5)	1,438	(17.1)
Years of education												
0–9	675	(46.6)	1,381	(49.7)	4,455	(46.6)	720	(53.5)	1,941	(48.3)	3,850	(45.7)
10–12	453	(31.3)	827	(29.8)	3,282	(34.3)	330	(24.5)	1,276	(31.7)	2,956	(35.1)
≥13	235	(16.2)	438	(15.8)	1,499	(15.7)	209	(15.5)	624	(15.5)	1,339	(15.9)
Missing	85	(5.9)	131	(4.7)	325	(3.4)	88	(6.5)	180	(4.5)	273	(3.2)
Smoking status												
Never smoker	742	(51.2)	1,385	(49.9)	5,334	(55.8)	669	(49.7)	2,154	(53.6)	4,638	(55.1)
Former smoker	402	(27.8)	768	(27.7)	2,457	(25.7)	346	(25.7)	1,040	(25.9)	2,241	(26.6)
Current smoker	167	(11.5)	333	(12.0)	888	(9.3)	167	(12.4)	433	(10.8)	788	(9.4)
Missing	137	(9.5)	291	(10.5)	882	(9.2)	165	(12.2)	394	(9.8)	751	(8.9)
Alcohol consumption												
Non-drinker	823	(56.8)	1,666	(60.0)	6,001	(62.8)	851	(63.2)	2,475	(61.6)	5,164	(61.3)
Drinker	547	(37.8)	939	(33.8)	3,039	(31.8)	418	(31.0)	1,325	(33.0)	2,782	(33.0)
Missing	78	(5.4)	172	(6.2)	521	(5.4)	78	(5.8)	221	(5.5)	472	(5.6)
Body mass index, kg/m^2^												
≤18.4	105	(7.3)	217	(7.8)	616	(6.4)	123	(9.1)	299	(7.4)	516	(6.1)
18.5–24.9	953	(65.8)	1,766	(63.6)	6,460	(67.6)	833	(61.8)	2,595	(64.5)	5,751	(68.3)
≥25.0	296	(20.4)	631	(22.7)	2,049	(21.4)	294	(21.8)	881	(21.9)	1,801	(21.4)
Missing	94	(6.5)	163	(5.9)	436	(4.6)	97	(7.2)	246	(6.1)	350	(4.2)
Treatment for any disease												
Without any disease	365	(25.2)	612	(22.0)	2,242	(23.4)	295	(21.9)	882	(21.9)	2,042	(24.3)
With any disease	957	(66.1)	1962	(70.7)	6,614	(69.2)	922	(68.4)	2,840	(70.6)	5,771	(68.6)
Missing	126	(8.7)	203	(7.3)	705	(7.4)	130	(9.7)	299	(7.4)	605	(7.2)
Physical strength^a^												
Normal	972	(67.1)	1,801	(64.9)	6,760	(70.7)	808	(60.0)	2,652	(66.0)	6,073	(72.1)
Low	301	(20.8)	606	(21.8)	1,685	(17.6)	344	(25.5)	862	(21.4)	1,386	(16.5)
Missing	175	(12.1)	370	(13.3)	1,116	(11.7)	195	(14.5)	507	(12.6)	959	(11.4)
Cognitive function^b^												
Normal	836	(57.7)	1,605	(57.8)	5,964	(62.4)	727	(54.0)	2,390	(59.4)	5,288	(62.8)
Decline	519	(35.8)	992	(35.7)	2,991	(31.3)	525	(39.0)	1,376	(34.2)	2,601	(30.9)
Missing	93	(6.4)	180	(6.5)	606	(6.3)	95	(7.1)	255	(6.3)	529	(6.3)
Geriatric depression scale												
0–4 (not depressed)	798	(55.1)	1,559	(56.1)	6,034	(63.1)	686	(50.9)	2,245	(55.8)	5,460	(64.9)
5–15 (depressed)	392	(27.1)	765	(27.5)	1,962	(20.5)	420	(31.2)	1,075	(26.7)	1,624	(19.3)
Missing	258	(17.8)	453	(16.3)	1,565	(16.4)	241	(17.9)	701	(17.4)	1,334	(15.8)
Instrumental ADL^c^												
Independent	1,051	(72.6)	2,015	(72.6)	7,201	(75.3)	893	(66.3)	3,026	(75.3)	6,348	(75.4)
Dependent	294	(20.3)	576	(20.7)	1,774	(18.6)	350	(26.0)	744	(18.5)	1,550	(18.4)
Missing	103	(7.1)	186	(6.7)	586	(6.1)	104	(7.7)	251	(6.2)	520	(6.2)

ADL, activities of daily living.

^a^Consists of 5 questions on participants’ self-reported physical strength. Participants who missed ≥3 points were regarded as being “low”.

^b^Consists of 3 questions on participants’ self-reported cognitive function. Participants who missed ≥1 point were regarded as showing a “decline”.

^c^Consists of 5 questions on participants’ self–reported instrumental ADL, such as use of public transport and management of money. Participants who missed ≥1 point were regarded as being “dependent”.

The total observation time was 36,619 person-years (average, 2.7 years/participant). Of the 13,786 participants, 1,203 cases (8.7%) of functional disability, 90 cases (0.7%) of movement out of the municipality, and 335 cases (2.4%) of death were recorded. The main results of our research are shown in Table 2, with a description of the hazard ratios of each bathing frequency group, analyzed by both crude and multivariate-adjusted models. In the multivariate-adjusted model, compared with the low-frequency group, the hazard ratios of the moderate- and high-frequency groups were 0.91 (95% confidence interval [CI], 0.75–1.10) and 0.72 (95% CI, 0.60–0.85) in summer and 0.90 (95% CI, 0.76–1.07) and 0.71 (95% CI, 0.60–0.84) in winter. Significant risk reduction was seen in the high-frequency group in both summer and winter. The hazard ratios were almost the same in summer and winter.

**Table 2.** Hazard ratios of functional disability onset according to the frequency of tub bathing in summer and in winter

	Frequency of tub bathing, times/week	Crude model			Multivariate-adjusted model^a^		
	
		HR	95% CIs	*P*-value	HR	95% CIs	*P*-value
Summer	0–2	reference			reference		
	3–6	1.00	(0.83–1.21)	0.995	0.91	(0.75–1.10)	0.323
	≥7	0.64	(0.54–0.75)	<0.001	0.72	(0.60–0.85)	<0.001

Winter	0–2	reference			reference		
	3–6	0.66	(0.56–0.78)	<0.001	0.90	(0.76–1.07)	0.246
	≥7	0.41	(0.35–0.48)	<0.001	0.71	(0.60–0.84)	<0.001

ADL, activities of daily living; HR, hazard ratio; CIs, confidence intervals.

^a^Adjusted for age, sex, marital status, employment, equivalized income, years of education, smoking status, alcohol consumption, body mass index, treatment for any disease, physical strength, cognitive function, depression, and instrumental ADL.

The results from the sensitivity analysis are shown in Table 3. After the exclusion of individuals whose follow-up period was less than 1 year, the inverse association between bathing frequency and functional disability onset remained consistent. Subgroup analysis results are presented in eTable 1, eTable 2, eTable 3, eTable 4, eTable 5, eTable 6, and eTable 7. The results were mostly consistent among subgroups.

**Table 3.** Sensitivity analysis: hazard ratios of functional disability onset, after the exclusion of participants whose follow-up period was <1 year

	Frequency of tub bathing, times/week	Multivariate-adjusted model^a^		

		HR	95% CIs	*P*-value
Summer	0–2	reference		
	3–6	0.93	(0.73–1.17)	0.517
	≥7	0.75	(0.61–0.93)	0.007

Winter	0–2	reference		
	3–6	0.94	(0.76–1.17)	0.592
	≥7	0.72	(0.59–0.89)	0.002

ADL, activities of daily living; HR, hazard ratio; CIs, confidence intervals.

^a^Adjusted for age, sex, marital status, employment, equivalized income, years of education, smoking status, alcohol consumption, body mass index, treatment for any disease, physical strength, cognitive function, depression, and instrumental ADL.

## DISCUSSION

Our main finding is that individuals who frequently took baths in a bathtub were less likely to be functionally disabled after adjustment for potential confounders. A seasonal difference in the association was not seen in the multivariate-adjusted model. The results from the sensitivity analysis weaken the possibility of a reverse-causation bias because potentially vulnerable individuals might be functionally disabled earlier. In addition, the results from the subgroup analysis imply that the association between exposure and outcome is consistent regardless of sex, age, or baseline health status.

Two pathways may explain the association between bathing and older people’s health status. First, tub bathing promotes good sleep^26^^,^^27^ and decreases sympathetic nerve activity.^28^ These changes may be beneficial for the mental status of older people and may prevent depression or cognitive function decline. Second, tub bathing raises the body temperature,^29^ which leads to increased expression of heat shock proteins (HSPs).^30^ HSPs have cytoprotective, anti-apoptotic, and anti-inflammatory effects.^31^ Additionally, HSPs are believed to play therapeutic roles in type 2 diabetes mellitus^32^ and Alzheimer’s disease.^33^ Thus, HSPs may improve or maintain the health conditions of older people. In Japan, the direct causes of older people’s functional disability are mainly dementia, cerebrovascular disease, arthrosis, fracture, and malignancy.^34^ The relationship between tub bathing and these diseases should be researched in a future study.

Our study results are consistent with those obtained in the aforementioned Finnish study,^14^ which indicated a robust negative association between sauna bathing frequency and cardiovascular and all-cause mortality, as well as several Japanese cross-sectional studies^10^^–^^12^ reporting a positive association between tub bathing frequency and self-rated health, which is a good predictor of functional disability in older people.^35^^,^^36^ Our results are also consistent with the only Japanese longitudinal study to examine this issue,^13^ which found a negative association between tub bathing and functional disability onset, although that study had a smaller sample size than ours and specifically targeted outpatients.

Strengths of our work include a large sample size, the enrollment of a general population in different regions in Japan, and the use of appropriate statistical methodology to adequately control for confounders. There are also several limitations. First, the certification of need for care did not always reflect functional disability. However, because this misclassification was expected to occur equally in each exposure group, we might actually have underestimated the association between exposure and outcome.^37^ Second, because we surveyed only tub bathing frequency, other types of bathing (ie, sauna or shower) were not taken into account, which limits the generalizability of our study when our findings are applied to people living in cultures without tub bathing. These issues should be investigated in a future study. Third, we could not examine the safety or risks of tub bathing in this study design. Sudden death or accidents during bathing are often reported in Japanese society.^38^^,^^39^ Therefore, safety issues related to tub bathing are important and should be studied appropriately. The relationship between bathing habit and mortality should be researched in a subsequent study, to take account of the risk of bathing-related death. Additionally, if our results are applied to clinical practice or recommended as an intervention, careful attention should be paid to safety. Considerations include advice for people with fever or excessive hypertension to avoid bathing^39^ and a reminder that the bathroom and dressing room should be warm.^40^ Lastly, we could not completely eliminate the reverse-causation bias, even after controlling for confounders and performing sensitivity analysis, because healthier people might bathe more frequently. However, this limitation is inevitable in an observational study.

In conclusion, our study indicates that a high frequency of tub bathing is associated with lower onset of functional disability among Japanese older adults. Further studies investigating the mechanisms linking tub bathing and older people’s health are warranted.

## APPENDIX A. SUPPLEMENTARY DATA

eFigure 1. Original question on tub bathing frequency (in Japanese)

eTable 1. Subgroup analysis: hazard ratios of functional disability onset stratified by sex

eTable 2. Subgroup analysis: hazard ratios of functional disability onset stratified by age

eTable 3. Subgroup analysis: hazard ratios of functional disability onset stratified by treatment for any disease

eTable 4. Subgroup analysis: hazard ratios of functional disability onset stratified by physical strength

eTable 5. Subgroup analysis: hazard ratios of functional disability onset stratified by cognitive function

eTable 6. Subgroup analysis: hazard ratios of functional disability onset stratified by depression

eTable 7. Subgroup analysis: hazard ratios of functional disability onset stratified by instrumental ADL
