# Supplementary material for: Bathing Frequency and Onset of Functional Disability Among Japanese Older Adults: A Prospective 3-Year Cohort Study From the JAGES
Source: J Epidemiol. 2019 Dec 5;29(12):451–6. doi: 10.2188/jea.JE20180123 (PMC6859082; doi:10.2188/jea.JE20180123)
Supplement: Supplementary file 1 [file je-29-451-s001.pdf]

Appendix

eFigure 1. Original question on tub bathing frequency (in Japanese)

【問 26】　お風呂（浴槽の湯につかる）はどのくらいの頻度で入りますか。

夏：週（　　　　　　　　　）回、　冬：週（　　　　　　　　　）回

eTable 1. Subgroup analysis: hazard ratios of functional disability onset stratified by sex

	Frequency of tub bathing,	Male[a]			Female[a]		
	times/week	HR	95% CIs	P-value	HR	95% CIs	P-value
Summer	0–2	reference			reference		
	3–6	1.02	(0.76–1.36)	0.923	0.83	(0.65–1.07)	0.159
	≥7	0.78	(0.59–1.02)	0.066	0.68	(0.54–0.85)	0.001
Winter	0–2	reference			reference		
	3–6	1.00	(0.77–1.31)	0.977	0.85	(0.68–1.06)	0.149
	≥7	0.69	(0.53–0.90)	0.005	0.71	(0.57–0.90)	0.003

ADL, activities of daily living; CIs, confidence intervals; HR, hazard ratio.

[a] Adjusted for age, marital status, employment, equivalized income, years of education, smoking status, alcohol consumption, body mass index, treatment for any disease, physical strength, cognitive function, depression, and instrumental ADL.

eTable 2. Subgroup analysis: hazard ratios of functional disability onset stratified by age

	Frequency of tub bathing, times/week	Age 65–74[a]			Age ≥75[a]		
		HR	95% CIs	P-value	HR	95% CIs	P-value
Summer	0–2	reference			reference		
	3–6	1.18	(0.78–1.78)	0.426	0.89	(0.72–1.11)	0.308
	≥7	0.87	(0.60–1.27)	0.478	0.69	(0.57–0.84)	<0.001
Winter	0–2	reference			reference		
	3–6	0.79	(0.53–1.16)	0.220	0.87	(0.72–1.05)	0.141
	≥7	0.60	(0.42–0.87)	0.007	0.67	(0.56–0.81)	<0.001

ADL, activities of daily living; CIs, confidence intervals; HR, hazard ratio.

[a] Adjusted for sex, marital status, employment, equivalized income, years of education, smoking status, alcohol consumption, body mass index, treatment for any disease, physical strength, cognitive function, depression, and instrumental ADL.

eTable 3. Subgroup analysis: hazard ratios of functional disability onset stratified by treatment for any disease

	Frequency of tub bathing,	Without any disease[a]			With any disease[a]		
	times/week	HR	95% CIs	P-value	HR	95% CIs	P-value
Summer	0–2	reference			reference		
	3–6	1.08	(0.66–1.78)	0.760	0.92	(0.73–1.15)	0.452
	≥7	0.61	(0.39–0.94)	0.027	0.74	(0.60–0.90)	0.003
Winter	0–2	reference			reference		
	3–6	0.60	(0.38–0.94)	0.027	0.97	(0.80–1.19)	0.778
	≥7	0.45	(0.29–0.68)	<0.001	0.75	(0.62–0.91)	0.004

ADL, activities of daily living; CIs, confidence intervals; HR, hazard ratio.

[a] Adjusted for age, sex, marital status, employment, equivalized income, years of education, smoking status, alcohol consumption, body mass index, physical strength, cognitive function, depression, and instrumental ADL.

eTable 4. Subgroup analysis: hazard ratios of functional disability onset stratified by physical strength

	Frequency of tub bathing, times/week	Normal Physical Strength[a]			Low Physical Strength[a]		
		HR	95% CIs	P-value	HR	95% CIs	P-value
Summer	0–2	reference			reference		
	3–6	0.75	(0.56–1.00)	0.052	0.99	(0.73–1.33)	0.923
	≥7	0.58	(0.45–0.74)	<0.001	0.78	(0.59–1.03)	0.077
Winter	0–2	reference			reference		
	3–6	0.77	(0.58–1.00)	0.052	0.91	(0.70–1.18)	0.455
	≥7	0.60	(0.46–0.77)	<0.001	0.72	(0.56–0.93)	0.011

ADL, activities of daily living; CIs, confidence intervals; HR, hazard ratio.

[a] Adjusted for age, sex, marital status, employment, equivalized income, years of education, smoking status, alcohol consumption, body mass index, treatment for any disease, cognitive function, depression, and instrumental ADL.

eTable 5. Subgroup analysis: hazard ratios of functional disability onset stratified by cognitive function

	Frequency of tub bathing, times/week	Cognitive function Normal[a]			Cognitive function Decline[a]		
		HR	95% CIs	P-value	HR	95% CIs	P-value
Summer	0–2	reference			reference		
	3–6	0.84	(0.62–1.13)	0.239	1.03	(0.78–1.35)	0.843
	≥7	0.69	(0.53–0.90)	0.005	0.77	(0.60–0.99)	0.040
Winter	0–2	reference			reference		
	3–6	0.77	(0.58–1.01)	0.055	0.99	(0.78–1.26)	0.930
	≥7	0.69	(0.53–0.89)	0.005	0.71	(0.56–0.90)	0.004

ADL, activities of daily living; CIs, confidence intervals; HR, hazard ratio.

[a] Adjusted for age, sex, marital status, employment, equivalized income, years of education, smoking status, alcohol consumption, body mass index, treatment for any disease, physical strength, depression, and instrumental ADL.

eTable 6. Subgroup analysis: hazard ratios of functional disability onset stratified by depression

	Frequency of tub bathing, times/week	Not Depressed[a]			Depressed[a]		
		HR	95% CIs	P-value	HR	95% CIs	P-value
Summer	0–2	reference			reference		
	3–6	1.08	(0.80–1.47)	0.610	0.86	(0.62–1.18)	0.352
	≥7	0.68	(0.52–0.90)	0.007	0.85	(0.63–1.14)	0.276
Winter	0–2	reference			reference		
	3–6	0.89	(0.68–1.18)	0.430	0.98	(0.74–1.30)	0.894
	≥7	0.64	(0.49–0.84)	0.001	0.83	(0.63–1.09)	0.176

ADL, activities of daily living; CIs, confidence intervals; HR, hazard ratio.

[a] Adjusted for age, sex, marital status, employment, equivalized income, years of education, smoking status, alcohol consumption, body mass index, treatment for any disease, physical strength, cognitive function, and instrumental ADL.

eTable 7. Subgroup analysis: hazard ratios of functional disability onset stratified by instrumental ADL

	Frequency of tub bathing, times/week	Independent in instrumental ADL[a]			Dependent in instrumental ADL[a]		
		HR	95% CIs	P-value	HR	95% CIs	P-value
Summer	0–2	reference			reference		
	3–6	1.02	(0.78–1.34)	0.871	0.78	(0.57–1.05)	0.100
	≥7	0.68	(0.53–0.87)	0.002	0.69	(0.53–0.91)	0.008
Winter	0–2	reference			reference		
	3–6	0.84	(0.66–1.08)	0.175	0.93	(0.72–1.22)	0.613
	≥7	0.63	(0.50–0.80)	<0.001	0.72	(0.56–0.92)	0.010

ADL, activities of daily living; CIs, confidence intervals; HR, hazard ratio.

[a] Adjusted for age, sex, marital status, employment, equivalized income, years of education, smoking status, alcohol consumption, body mass index, treatment for any disease, physical strength, cognitive function, and depression.
